# Functional Brain Activity Changes after 4 Weeks Supplementation with a Multi-Vitamin/Mineral Combination: A Randomized, Double-Blind, Placebo-Controlled Trial Exploring Functional Magnetic Resonance Imaging and Steady-State Visual Evoked Potentials during Working Memory

**DOI:** 10.3389/fnagi.2016.00288

**Published:** 2016-12-02

**Authors:** David J. White, Katherine H. M. Cox, Matthew E. Hughes, Andrew Pipingas, Riccarda Peters, Andrew B. Scholey

**Affiliations:** ^1^Centre for Human Psychopharmacology, School of Health Sciences, Swinburne University, Hawthorn, VICAustralia; ^2^Brain and Psychological Sciences Research Centre, School of Health Sciences, Swinburne UniversityHawthorn, VIC, Australia

**Keywords:** multi-vitamin, *nutrient*, functional magnetic resonance imaging (fMRI), steady-state visual evoked potentials, working memory

## Abstract

This study explored the neurocognitive effects of 4 weeks daily supplementation with a multi-vitamin and -mineral combination (MVM) in healthy adults (aged 18–40 years). Using a randomized, double-blind, placebo-controlled design, participants underwent assessments of brain activity using functional Magnetic Resonance Imaging (fMRI; *n* = 32, 16 females) and Steady-State Visual Evoked Potential recordings (SSVEP; *n* = 39, 20 females) during working memory and continuous performance tasks at baseline and following 4 weeks of active MVM treatment or placebo. There were several treatment-related effects suggestive of changes in functional brain activity associated with MVM administration. SSVEP data showed latency reductions across centro-parietal regions during the encoding period of a spatial working memory task following 4 weeks of active MVM treatment. Complementary results were observed with the fMRI data, in which a subset of those completing fMRI assessment after SSVEP assessment (*n* = 16) demonstrated increased BOLD response during completion of the Rapid Visual Information Processing task (RVIP) within regions of interest including bilateral parietal lobes. No treatment-related changes in fMRI data were observed in those who had not first undergone SSVEP assessment, suggesting these results may be most evident under conditions of fatigue. Performance on the working memory and continuous performance tasks did not significantly differ between treatment groups at follow-up. In addition, within the fatigued fMRI sample, increased RVIP BOLD response was correlated with the change in number of target detections as part of the RVIP task. This study provides preliminary evidence of changes in functional brain activity during working memory associated with 4 weeks of daily treatment with a multi-vitamin and -mineral combination in healthy adults, using two distinct but complementary measures of functional brain activity.

## Introduction

Micronutrients, such as B vitamins and minerals, are critical components of healthy physiological functioning, yet humans are dependent on dietary sources in maintaining an adequate supply. B vitamins are a group of water soluble organic molecules which act as cofactors in a multitude of cellular processes. These micronutrients are implicated in catabolic reactions which support energy metabolism (Depeint et al., 2006; Huskisson et al., 2007), in addition to anabolic pathways which drive synthesis of DNA/RNA, antioxidants and neurotransmitters, whilst also reducing homocysteine to methionine through roles in one carbon transfer cycles (for a detailed review, see Kennedy, 2016). While a healthy diet may be sufficient to maintain adequate supplies of these essential micronutrients, modern Western dietary patterns may lead to a significant proportion of the population not meeting recommendations for a range of micronutrients, including B vitamins (Troesch et al., 2012).

Looking beyond the prevention of physical illness related to deficiency in specific B vitamins, there is very little data exploring optimal micronutrient levels (Neufeld and Cameron, 2012; Kennedy, 2016). Recent work investigating micronutrient supplementation with a multi-vitamin/mineral preparation (MVM) in healthy individuals provides support for a distinction between optimal and sub-optimal micronutrient status (Pietrzik, 1985; Fletcher and Fairfield, 2002) in the absence of a clinical deficiency. For example, clinical trials investigating the effects of MVM in healthy adults have generally observed a positive impact on mood, with meta-analysis indicating significant benefits to subjective stress, sub-clinical psychiatric symptoms and a range of everyday mood dimensions (Long and Benton, 2013). Indeed, mood outcomes were assessed as part of the trial which generated the functional brain activity assessments described herein, also showing significant positive effects for aspects of everyday mood (White et al., 2015).

Given the prominent role of B vitamins in cellular functions which support brain function, it follows that manipulations of micronutrients such as B vitamins via MVM supplementation may impact neurocognitive function. Trials investigating potential effects of MVM supplementation on cognitive performance amongst healthy adults have not provided a wholly consistent picture, partially driven by insufficient research (Grima et al., 2012). The potential for enhancing cognitive function amongst healthy adult males was studied by Kennedy et al. (2010). Serial subtraction task performance completed as part of a cognitive demand battery was improved following 28-days MVM supplementation. In another study, females aged 25–50 years demonstrated significant cognitive performance benefits after 63 days of MVM supplementation using a multi-tasking assessment (Haskell et al., 2010). In contrast, the largest MVM supplementation trial to date investigating cognitive changes found no evidence supporting a positive effect of such treatment in a sample of male physicians aged 65 or older over a mean follow-up intervention duration of 8.5 years (Grodstein et al., 2013). These studies not only highlight the variability in findings when considering possible cognitive function benefits associated with MVM supplementation in healthy adults, but also the differences in micronutrient doses and duration of the MVM treatment, the treatment populations, and the methods for assessing cognitive performance (for example, Grodstein et al., 2013 assessed cognitive function by telephone interview).

Recently, a parallel line of inquiry has explored potential physiological changes associated with MVM supplementation. Amongst obese females aged 18–55, daily MVM supplementation with a preparation containing a large number of minerals in addition to vitamins over 26 weeks led to an increase in resting energy expenditure, along with reductions in fat mass, body weight, and cholesterol (Li et al., 2010). Increased energy expenditure was again observed in a sample of healthy females aged 25–49 after MVM supplementation, in this case during cognitive task performance (Kennedy et al., 2016). Exploring both acute and acute-on-chronic effects of two MVM treatments, one higher dose and one lower dose with added Coenzyme Q10, Kennedy et al. (2016) reported this increase in energy expenditure during cognitive task performance after both the single acute dose and in a dose-dependent manner following 56 days of supplementation. The study also explored brain hemodynamic changes during completion of the cognitive task battery using functional near infrared spectroscopy (fNIRS), which revealed increased cerebral blood flow to prefrontal sites at the first acute assessment only with the lower MVM dose with Coenzyme Q10 and similar, albeit non-significant, trends in the high dose MVM treatment group. These hemodynamic changes were not accompanied by changes in cognitive function.

Functional Magnetic Resonance Imaging (fMRI) shares some of the properties of fNIRS but allows far better spatial resolution. Work from our laboratory has provided preliminary evidence of neurophysiological changes associated with MVM supplementation, exploring both acute doses and longer term supplementation using fMRI and Steady State Visual Evoked Potentials (SSVEP) – an electrophysiological technique with high temporal resolution (see below). In a pilot investigation of the acute effects of two MVM treatments, with or without guaraná, fMRI during completion of Rapid Visual Information Processing (RVIP) and Inspection Time tasks revealed increased blood oxygen level dependent (BOLD) response to active tasks for both MVM treatments compared to placebo (Scholey et al., 2013). This study also reported cognitive performance and mood benefits restricted to the MVM plus guaraná treatment.

Changes in functional brain activity have been reported for both acute and chronic MVM supplementation using SSVEP methodology. Before proceeding with the outcomes of this work, a brief introduction to the SSVEP technique is provided. The SSVEP has found applications in the study of basic visual processes and perception, brain-computer interfaces and higher order cognitive functions (for reviews, see Vialatte et al., 2010; Norcia et al., 2015). One SSVEP method for probing higher cognitive functions, referred to as Steady State Topography (Silberstein et al., 1990), explores SSVEP responses to a task-irrelevant diffuse 13 Hz sinusoidal flicker superimposed onto the visual field during cognitive task engagement. SSVEP amplitude reflects summed activity of pyramidal cells firing synchronously with the 13 Hz flicker, whilst SSVEP phase reflects latency differences between the visual flicker and the SSVEP response. This phase difference is argued to reflect cortico-cortical loop transmission time, which is linked with ongoing excitatory and inhibitory processes in the underlying cortical regions such that an increase in excitatory processes will manifest as an SSVEP phase advance (or reduced latency; Silberstein et al., 1995, 2000). Using this Steady State Topography method, fluctuations in SSVEP amplitude and phase have been linked with a range of cognitive processes (Silberstein et al., 2001; Kemp et al., 2002; Ellis et al., 2006), neurophysiological changes in clinical populations (Silberstein et al., 1998, 2000), and also to probe the neurocognitive effects of a number of psychopharmacological and nutritional manipulations (Kemp et al., 2004; Silberstein et al., 2011; Camfield et al., 2012), including MVM supplementation.

The pilot fMRI findings of Scholey et al. (2013) studying acute effects of MVM administration were extended using SSVEP recordings during completion of the A-X Continuous Performance Task (A-X CPT; White et al., 2016). This study found distinct changes in SSVEP response during the A-X CPT associated with MVM supplementation containing higher B vitamin and mineral doses and a second MVM with lower micronutrient doses but additional guaraná. After the high dose MVM treatment, SSVEP amplitude reduction and phase advance were observed in frontal electrode sites during the period between the probe and target stimuli, a pattern which was correlated with better task performance. In contrast, the low dose MVM with guaraná showed little evidence of any phasic task-related SSVEP fluctuations, instead causing a generalized shift toward greater phase advance, with a diffuse topographic pattern, consistent with the general excitatory actions of the caffeine content in the treatment. Macpherson et al. (2012) explored changes in functional brain activity related to MVM supplementation over 16 weeks in females aged 64–79 using SSVEP recordings. This trial tested the effects of supplementation with an MVM preparation, which also contained 19 plant extracts and three probiotics, with changes in SSVEP studied during completion of a spatial working memory task. The study observed greater SSVEP phase lag (increased latency) during the retrieval component of the spatial working memory task, a pattern of change which was correlated with improved task performance post-treatment.

To summarize, micronutrients are essential cofactors in a host of cellular processes critical to healthy physiological and neurocognitive functioning, with an increasing effort to understand whether intake other than in the context of avoiding clinical deficiency may enhance function in otherwise healthy individuals. Where studies have considered behavioral outcomes of MVM interventions in healthy adults, results indicate potential benefits to mood, with support, though less consistent, for cognitive performance benefits. There is also evidence to suggest MVM supplementation may influence energy metabolism, and possibly more specific neurophysiological functions. In studies assessing task-related SSVEP changes following MVM administration, the technique appears sensitive to potential changes in functional brain activity related to MVM supplementation, whilst the pilot data using fMRI methods warrant further investigation. To this end, the current study aimed to utilize these complementary methods, fMRI and SSVEP, to assess functional brain activity in order to explore any potential relationship between MVM supplementation and brain function in healthy adults.

## Materials and Methods

We studied functional imaging outcomes, employing a randomized, double-blind, placebo controlled design to investigate the effects of 4 weeks daily supplementation with a MVM combination. The intervention consisted of a micronutrient preparation containing all eight B vitamins, in addition to Vitamin C and the minerals calcium, magnesium and zinc (further detailed below). The overall trial methods and outcomes relating to mood and blood biomarkers have been published elsewhere (White et al., 2015). The study was approved by the Swinburne University Human Research Ethics Committee (Ref SUHREC 2012/164) and was registered with the Australian New Zealand Clinical Trials Registry (ACTRN12612001043820).

### Participants

The study enrolled 58 healthy adult participants aged between 18 and 40 years of age (Mean age = 25.82 years, *SD* = 4.87), who were recruited from the local community via local advertisements. Participants were free of psychiatric or serious physical illnesses and had not taken medication (with the exception of the contraceptive pill or routine medications for benign conditions), herbal extracts, vitamin supplements or illicit drugs within 4 weeks prior to enrolment and for study duration. These participants completed one of three functional brain imaging assessment streams (further detailed in Supplementary Figure 3): both SSVEP and fMRI (*n* = 16, eight females, one additional participant withdrew consent prior to follow-up assessment), SSVEP assessment alone (*n* = 23, 12 female), and fMRI assessment alone (*n* = 16, eight females, with two additional participants who withdrew consent prior to follow-up assessment). Those who completed both SSVEP and fMRI assessment did so on the same testing day, with SSVEP recordings undertaken prior to fMRI assessment.

### Procedure

Participants attended an initial screening and familiarization visit, followed by baseline and post-treatment assessment sessions conducted 4 weeks apart. Participants were given an opportunity to practice each of the cognitive tasks at the screening visit, with SSVEP and fMRI assessment of functional brain activity performed at baseline and 4 weeks post-treatment. Randomization was conducted by a disinterested third party, with stratified randomization used to balance gender within each functional imaging assessment stream. The active and placebo treatments were effervescent tablets matched for color and flavor, prepared by Bayer AG (Basel, Switzerland). The active treatment, containing high doses of B vitamins, in addition to zinc, calcium, magnesium and vitamin C, is commercially available as Berocca^®^ Performance (detailed in **Table 1** below). Participants were instructed to take one tablet daily with breakfast, dissolved in at least 200 mL of water. Participants returned after 4 weeks of supplementation having not taken a treatment on the day of their return visit. Compliance was determined by a count of returned treatments, whilst analysis of circulating levels of vitamin B6, Red Cell Folate and B12 showed increases post-treatment in the active treatment group, with significant reductions in homocysteine, confirming compliance and absorption (for details, see White et al., 2015).

**Table 1:** Micronutrient doses of the active MVM treatment.

Nutrient	Amount (mg)	% RDA/RDI	
		Males	Females
Vitamin C	500	556 (1111)	667 (1111)
Thiamine monophosphoric acid ester chloride	18.54	1545	1685
Riboflavin (vitamin B2)	15	1154	1364
Nicotinamide (B3/niacin)	50	313	357
Vitamin B5	23	460^∗^ (383^∗^)	460^∗^ (575^∗^)
Vitamin B6	10	769	769
Vitamin B12	0.01	417	417
Folic Acid (Vitamin B9)	0.4	100	100
Biotin (Vitamin B7)	0.15	500^∗^	500^∗^ (600^∗^)
Calcium	100	10	10
Magnesium	100	25	32
Zinc	10	91 (71)	125

*% RDA (RDI) is the percentage for each micronutrient of Recommended Dietary Allowances derived from Dietary References Intakes of the Institute of Medicine, National Academy of Sciences (US) and Recommended Daily Intake from the Nutrient Reference Values of the National Health and Medical Research Council (Australia) for healthy adults in the age range 19–30. Where a second number appears in brackets, the US and Australian reference values disagree, in which case US RDA’s are given first with Australian RDI’s in brackets. ^∗^Indicates the use of ‘Adequate Intakes’ where RDA/RDI could not be determined.*

### Functional MRI Acquisition

MRI scanning used a Siemens Tim Trio 3T MRI scanner equipped with a 32-channel head coil at Swinburne University of Technology, Melbourne, Australia. Functional MRI data was acquired during completion of an Inspection Time (IT) task and the RVIP task. At baseline and post-treatment assessments, the imaging protocol included a T1-weighted scan (3D MPRAGE; TR = 1900 ms, TE = 2.52 ms, flip angle = 9°, Field of View = 256 mm, 176 slices, 1 mm^3^ isotropic voxels), in addition to T2^∗^-weighted volumes obtained during completion of the two cognitive tasks using the same EPI acquisition parameters, with the first three volumes of each task run discarded (TR = 3000 ms, TE = 30 ms, flip angle = 90°, Field of View = 192 mm, 46 interleaved slices, 3 mm^3^ isotropic voxels). Both IT and RVIP tasks, described below, were variants of previously published task paradigms presented using Presentation^®^ software^1^. Participants viewed the task stimuli on a monitor through a mirror on the head coil.

#### Inspection Time Task Details

The IT task involved presentation of a stimulus with two vertical lines of varying lengths running perpendicular at either end of a horizontal line (white stimuli on a black background). The participant was then required to indicate which line was perceived as longer (forced choice, with two alternatives, via button box held in the right hand with an index finger (left) or middle finger (right) button press). The duration for which this stimulus remained visible, prior to the appearance of a mask (500 ms mask duration), varied from equally represented trials of 40, 60, 80, 100, and 120 ms (Waiter et al., 2008). Trials were preceded by a 600 ms fixation cross, with a variable inter-trial interval (ITI) comprising each 500 ms increment from 1880 to 3880 ms. Each ITI preceded the five stimulus durations equally, with 20 trials of each stimulus duration presented in a pseudorandom order per run of the task. Two 426-second runs (142 functional volumes), each of 100 trials, were conducted at each assessment visit.

#### Rapid Visual Information Processing Task Details

The RVIP task implemented was identical to that described in Neale et al. (2015). Briefly, single digits (white stimuli on a black background) were presented at a rate of 100 per minute, with the active task variant requiring participants to respond upon presentation of three consecutive odd or even digits, and a control task variant requiring detection of a single target digit not presented during the active variant (‘0’). Both task variants contained target sequences at a rate of four per 30 s. The two task variants alternated in a blocked design, with each 58.5-second block preceded by a 12-second rest/instruction block, which cued participants to the upcoming task requirements. Two runs of the task were completed at each assessment visit, with each run comprising three blocks of each task variant and seven rest/instruction blocks for a total of 435 s (145 functional volumes per run).

#### fMRI Data Processing and Analysis

All pre-processing and participant-level statistical analysis of fMRI data were performed using SPM8 (Wellcome Trust Centre for Neuroimaging, London, UK). Data pre-processing included the following steps: the image time-series were slice-time corrected referenced to the middle slice, then realigned to the first volume acquired. The T1 structural image acquired at the corresponding treatment visit was co-registered to a mean functional image created during realignment, then normalized to MNI T1 template released with SPM8. The parameters of this T1 transformation were subsequently applied to the realigned functional images. The normalized functional images were then smoothed with an 8 mm FWHM Gaussian filter.

Participant-level modeling for the IT task explored the parametric modulation of BOLD signal as a function of stimulus duration. Correct trial events were modeled as stick functions convolved with the canonical hemodynamic response function (HRF) supplied with SPM8. These correct events were parametrically modulated with a regressor coding trial presentation duration (longest durations had the highest numbers) that was orthogonalized to the event regressor; the parametric modulator expresses how well BOLD fluctuations not explained by the average response to all stimulus onsets covaries with stimulus presentation length. Finally, motion correlated BOLD signal variance was modeled by including the motion realignment parameters as covariates of no interest.

At the group level of analysis, the parametric modulation effect showed no statistically significant effects at baseline (see Supplementary Data Section 2). In the absence of reliable task effects at baseline, this task was not pursued for potential effects of treatment.

Participant-level modeling for the RVIP task modeled active and control variants of the task using separate boxcar functions defined by the onset and duration of each task block that were convolved with the canonical HRF, with the motion parameters included as nuisance regressors. Analysis of treatment effects on RVIP task-related functional brain activity adopted a region-of-interest (ROI) approach, deriving functional ROIs from an independent sample of healthy participants matching eligibility criteria for the study. The details of the analysis generating the five ROIs used are described in Supplementary Data Section 1 (see Supplementary Table 1 and Supplementary Figure 1). The five ROIs identified by this analysis were localized to a left and right parietal cluster, a left middle frontal cluster, the left insula and a cluster comprising bilateral supplementary motor areas. Mean contrast estimates obtained from the active task minus control task contrast were extracted from the five ROIs, using the MarsBaR toolbox^2^ (Brett et al., 2002). Given the demands of the control task largely match the attentional and sensorimotor demands of the active variant, this contrast largely isolates working memory demands required by the active variant of the task. These mean contrast estimates obtained at the post-treatment visit formed the input for an analysis of covariance (ANCOVA) examining the main effect of treatment group as a between-group factor, with pre-treatment baseline estimates included as a covariate using SPSS for Windows (Version 23; SPSS Inc., Chicago, IL, USA). Comparisons were thresholded for significance using False Discovery Rate correction with *q* = 0.1 in order to control for multiple comparisons across the five ROIs (Benjamini and Hochberg, 1995).

### Steady-State Visually Evoked Potential Recordings

Two cognitive tasks, one continuous performance task and one spatial working memory task, were completed during recordings of the SSVEP. Acquisition and pre-processing of the SSVEP followed an identical procedure to that described in a recent study exploring the acute effects of MVM administration, both with and without guaraná (White et al., 2016). Briefly, recordings were acquired from 60 scalp electrodes, positioned according to the extended 10-10 system using a Quick-Caps electrode cap and Synamps^2^ amplifiers with Scan 4.3 Software (Neuroscan, Inc., Abbotsford, VIC, Australia). An electrode positioned between Cz and Cpz acted as the reference, with the ground electrode positioned between Fz and Fpz. Recordings were also taken from left and right mastoids, with data subsequently re-referenced to linked mastoids off-line. The SSVEP was elicited through goggles using LED arrays emitting a 13 Hz sinusoidal flicker, which was superimposed over the visual field with half-silvered mirrors subtending a horizontal angle of 160° and vertical angle of 90°.

#### SSVEP Cognitive Tasks: A-X Continuous Performance Task

The details of the AX-CPT used in the present study are identical to that described in the previous investigation of the acute effects of MVM administration (White et al., 2016). The task presents single letters at a rate of approximately 34 per minute, and involves two variants: one active task in which participants respond to a target sequence of ‘X’ preceded by ‘A’ amongst a pseudo-random sequence of letters, and a control task in which participants respond to a target letter ‘E’ amongst a predictable repeated sequence of letters (‘A’ through ‘E’). For both variants 200 stimuli, including 40 target responses, were presented across two recording runs of approximately 3 min each separated by a short break.

#### SSVEP Cognitive Task: Spatial Working Memory

The spatial working memory task adopted for the current study was based on a delayed match-to-sample paradigm with a history of use in studying the functional imaging correlates of spatial working memory (Jonides et al., 1993), which has previously been used to explore age-related changes in SSVEP response (Macpherson et al., 2014), in addition to changes associated with MVM supplementation in healthy older females (Macpherson et al., 2012). Trials presented either two or three white dots on a black background for 500 ms (encoding period), followed by presentation of a fixation cross for 3000 ms (maintenance period), after which an empty circle appeared for 1800 ms (probe). During this probe period, participants responded as to whether the location of the circle enclosed an encoded location of the dot stimuli, with a right button press indicating a match and a left button press indicating a new location. The control variant of the task matched sensorimotor demands of the task, removing the working memory load by retaining the dot stimuli on-screen throughout the duration of each trial, such that the probe period required a response as to whether the circle stimuli either surrounded a dot (right button press) or did not enclose a dot (left button press). Active and control task variants were completed in separate recording runs, comprising 40 trials separated by a 1000 ms fixation period, half of which presented two dots during encoding and half three dots. To ensure a sufficient number of correct trials for analysis, two runs of the active task variant were completed, resulting in 80 trials for the active task per visit and a single run of 40 trials for the control variant.

#### SSVEP Data Processing and Analysis

All aspects of the SSVEP signal processing were identical to that described in White et al. (2016), with the 13 Hz SSVEP signal extracted from ongoing recordings using established routines implemented in in-house software (BrainSci; Silberstein, 1995b) and statistical analysis and mapping using custom MATLAB scripts (The Mathworks Inc., Natick, MA, USA). In order to control for inter-individual differences in SSVEP amplitude and phase responses, the active task variants of both AX-CPT and SWM task paradigms were normalized to the corresponding control task variant at the baseline visit. For the A-X CPT task, mean SSVEP amplitude and phase were calculated for the 250 ms period following the cue stimulus (‘A’), the 1500 ms hold period following, and the 1000 ms from target appearance (‘X’). For the SWM task, mean SSVEP amplitude and phase were calculated for the 500 ms encoding period, the 3000 ms maintenance period, and the 1000 ms from presentation of the probe stimulus. As only correct trials were included in analyses, one further participant was excluded from analysis of A-X CPT, due to incorrect task performance.

Both A-X CPT and SWM tasks were studied across the entire SSVEP sample at baseline to characterize the SSVEP response to task completion pre-treatment. For each of the three averaged task components, this involved direct comparison between the active and control task variant for both tasks. Subsequently, potential changes in SSVEP response from baseline to post-treatment were investigated, contrasting the active task SSVEP response at each visit for each treatment group separately. The SSVEP response is studied in terms of amplitude and phase, necessitating an alternate statistical approach to that of behavioral and fMRI data. Comparisons were conducted using Hotelling’s T^2^, the bivariate analog to a paired *T*-test, which tests for differences in the mean vector comprising the complex numbers quantifying the SSVEP amplitude and phase. Adjustment for multiple comparisons followed previous research to use the Steady State Topography method, in which spatial principal component analysis of SSVEP data indicating five independent components can account for over 95% of spatial variance (Silberstein and Cadusch, 1992), thus the alpha level for SSVEP analysis was adjusted to 1% (adjusted *p* = 0.05/5). In the event of significant changes, the association between these SSVEP changes and behavioral performance changes from baseline to post-treatment were also explored through correlations in order to assess any behavioral correlates of these changes (that is, the difference in SSVEP response pre- to post-treatment was correlated with the change in performance between these assessment visits).

### Analysis of Behavioral Performance

Behavioral performance on cognitive tasks was measured in terms of accuracy and mean response time for the active variant of each of the four cognitive tasks. Task accuracy was operationalized as the percentage of correct responses for IT and spatial working memory tasks and the mean number correct target detections per task run in the case of the RVIP (out of 24 per run) and A-X CP tasks (out of 20 per run). To investigate potential treatment-related changes in behavioral performance, task accuracy and response times at the post-treatment visit formed the input for an analysis of covariance (ANCOVA) examining the main effect of treatment group as a between-group factor, with pre-treatment baseline estimates included as a covariate using SPSS for Windows (Version 23; SPSS Inc., Chicago, IL, USA). Criteria for significance across behavioral outcomes was set to *p* < 0.05.

### Analysis Populations

The analysis population for each outcome required complete cases (55 of the 58 randomized completed follow-up assessments), with the following additional criteria for each type of outcome: (a) behavioral performance data: primary analysis on complete cases with additional exclusion of outliers identified during data-screening conducted while blind to treatment, (b) for fMRI: primary analysis on complete datasets, with follow-up sensitivity analyses excluding behavioral outliers identified during blind data-screening, (c) for SSVEP data: primary analysis was conducted on complete cases with additional exclusion of datasets with excessive artifact, as defined by an arbitrary threshold described below, with follow-up analyses excluding behavioral outliers.

Prior to unblinding, behavioral outcomes were screened for outliers, in which behavioral performance approximating chance levels was observed for five participants on the IT task, who were subsequently excluded from analysis. Poor performance was also observed for a single participant on the RVIP task, three participants on the A-X CPT, and a single participant on the Spatial Working Memory task who were excluded from analysis of these behavioral outcomes. An additional three participants were missing behavioral data for the RVIP task due to a technical fault with the response device in the scanner, and were thus missing from analysis of behavioral results. Excessive SSVEP artifact was defined by a circular statistic exceeding 0.2 in 10 or more electrodes for a given task variant (described in Silberstein, 1995a), calculated for the first 120 s of each task run. This threshold resulted in the exclusion of three datasets from the Spatial Working Memory task, and no datasets from the A-X CPT. As fMRI data was acquired from two cohorts in which testing procedures differed, these groups were analyzed separately. As previously reported, assessment of functional brain activity using SSVEP recordings during A-X CPT and Spatial Working Memory tasks significantly decreased subjective ratings of mental stamina, concentration and alertness, while significantly increasing mental fatigue (further detailed in White et al., 2016), further emphasizing the need to consider the cohort in which fMRI was completed after SSVEP assessment separately.

## Results

### Behavioral Performance

Behavioral performance on the cognitive tasks completed during monitoring of functional brain activity using SSVEP and fMRI methods did not significantly differ between treatment groups post-treatment, when controlling for baseline scores. Mean performance on MRI-based tasks tended to be poorer at both visits in the fatigued sample, though the two testing cohorts did not significantly differ. These results are summarized in **Table 2** below.

**Table 2:** Behavioral performance on cognitive tasks.

Cohort	Task/outcome	Treatment	*M* (adj)	*SE*	*F*	*p*
SSVEP	A-X CPT – Hits (/20)	Plac (*n* = 18)	18.72	0.41	0.16	0.691
		MVM (*n* = 18)	18.95	0.41		
	A-X CPT – RT	Plac (*n* = 18)	357	10.54	3.07	0.089
		MVM (*n* = 18)	331	10.54		
	Spatial Working Memory – Accuracy	Plac (*n* = 20)	76.62	1.57	0.01	0.924
		MVM (*n* = 18)	76.40	1.65		
	Spatial Working Memory – RT	Plac (*n* = 20)	631	23	1.26	0.269

		MVM (*n* = 18)	669	24		
fMRI: Fatigued	IT – Accuracy	Plac (n = 5)	92.56	2.97	2.25	0.168
		MVM (*n* = 7)	86.17	2.41		
	IT – RT	Plac (*n* = 5)	471	35	0.40	0.545
		MVM (*n* = 7)	442	29		
	RVIP – Hits (/24)	Plac (n = 6)	14.43	1.60	0.82	0.388
		MVM (*n* = 6)	16.49	1.60		
	RVIP – RT	Plac (*n* = 6)	487	24	0.12	0.735
		MVM (*n* = 6)	476	24		

fMRI: Non-fatigued	IT – Accuracy	Plac (n = 6)	94.97	0.92	0.41	0.537
		MVM (*n* = 7)	94.17	0.85		
	IT – RT	Plac (n = 6)	452	34	0.23	0.644
		MVM (*n* = 7)	430	31		
	RVIP – Hits (/24)	Plac (n = 8)	19.29	0.66	1.28	0.278
		MVM (*n* = 8)	18.21	0.66		
	RVIP – RT	Plac (n = 8)	464	20	0.20	0.663
		MVM (*n* = 8)	477	20		

*Mean (and Standard Error) accuracy and response time for cognitive tasks completed during functional brain activity assessment at post-treatment assessment, adjusted for baseline performance. F- and p-values correspond to ANCOVA model testing main effect of treatment group on post-treatment performance, controlling for baseline performance. M (adj), Baseline adjusted estimated marginal means of post-treatment performance; RT, response time;Hits, mean target detections; MVM, multi-vitamin and mineral treatment group; Plac, Placebo; ANCOVA results reported for main effect of treatment group.*

### fMRI

#### Task Effects

Whole brain voxel-wise patterns of task-related activity were studied at baseline, in order to confirm that performance of the RVIP and IT tasks elicited activity consistent with previous research, also justifying the regions of interest identified from the independent sample for use in exploring treatment-related effects during RVIP task performance. The IT task failed to show reliable task effects, when exploring parametric modulation of the BOLD signal by stimulus presentation length (cf. Deary et al., 2004; Waiter et al., 2008), and as such was not pursued further for effects of treatment. The RVIP task showed results consistent with previous functional imaging studies of this task (Coull et al., 1996; Lawrence et al., 2003; Neale et al., 2015) and the ROIs defined from the independent sample, including large regions of bilateral parietal and frontal cortex. The outcomes of these analyses are further detailed in Supplementary Data Section 2 (see Supplementary Table 2 and Supplementary Figure 2).

#### RVIP Post-treatment Effects

**FIGURE 1:**
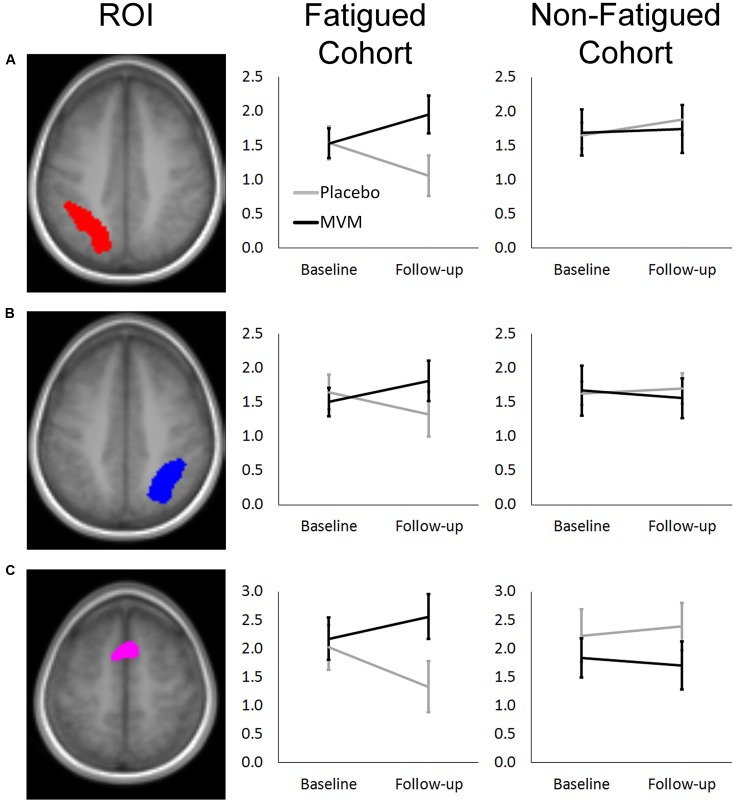
**Mean RVIP task contrast estimates extracted at baseline and post-treatment for fatigued (left) and non-fatigued (right) cohorts for (A)** Left Parietal, **(B)** Right Parietal, and **(C)** SMA regions of interest, where significantly greater task-related activity was observed in active treatment group post-treatment within the fatigued cohort. Error bars ±1 SE.

Amongst fatigued participants, who underwent SSVEP assessment prior to fMRI, mean contrast estimates derived from subtracting the control RVIP task variant from the active RVIP task variant were significantly higher post-treatment in the MVM treatment group compared to placebo, when controlling for baseline. This effect survived FDR correction across three ROIs: bilateral parietal lobe ROIs and the supplementary motor area ROI. Repeating this analysis with the exclusion of a single poor-performing participant (placebo group) showed the same pattern of results, with left parietal and SMA ROIs surviving FDR correction. In contrast, no significant differences between treatment groups were observed amongst the non-fatigued cohort of participants. These outcomes are detailed in **Table 3**, while **Figure 1** depicts mean contrast estimates for fatigued and non-fatigued groups pre- and post-treatment within the three ROIs for which significant differences between treatment groups were observed.

**Table 3:** Mean RVIP task-related contrast estimates post-treatment from the five regions of interest for each treatment group, adjusted for baseline, in both fatigued (*n* = 16) and non-fatigued cohorts (*n* = 16).

	ROI	Treatment	*M* (adj)	*SE*	*F*	*p*	η_p_^2^
**Fatigued**	Left Parietal	Plac	1.06	0.23	7.95	0.015 ^∗^	0.379
		MVM	1.96	0.23			
	Right Parietal	Plac	1.25	0.21	4.84	0.047 ^∗^	0.271
		MVM	1.89	0.21			
	Left Frontal	Plac	2.08	0.54	2.17	0.164	
		MVM	3.21	0.54			
	Supplementary Motor Area	Plac	1.40	0.29	7.35	0.018 ^∗^	0.361
		MVM	2.50	0.29			
	Left Insula	Plac	1.32	0.32	0.39	0.543	
		MVM	1.62	0.32			

**Non-fatigued**	Left Parietal	Plac	1.88	0.30	0.10	0.760	
		MVM	1.75	0.30			
	Right Parietal	Plac	1.69	0.26	0.13	0.726	
		MVM	1.56	0.26			
	Left Frontal	Plac	2.90	0.52	0.06	0.808	
		MVM	2.72	0.52			
	Supplementary Motor Area	Plac	2.39	0.44	1.22	0.290	
		MVM	1.70	0.44			
	Left Insula	Plac	1.63	0.40	0.21	0.655	
		MVM	1.37	0.40			

*ROI, region of interest; M (adj), baseline adjusted estimated marginal means;Plac, placebo group; MVM, multi-vitamin and mineral group; ANCOVA results reported for main effect of treatment group; ^∗^significant after FDR thresholding.*

**FIGURE 2:**
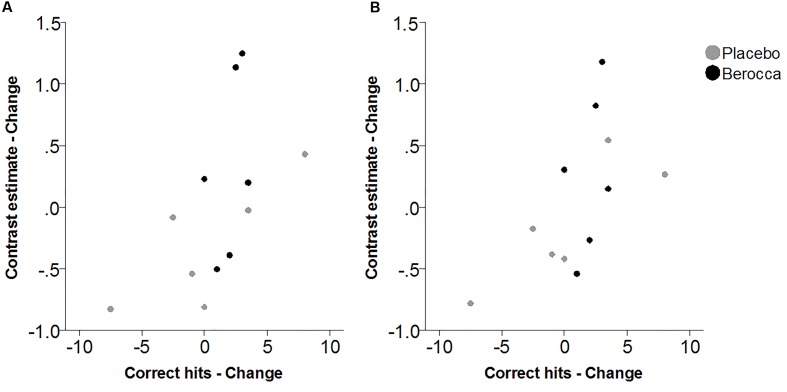
**Scatterplots of association between change in RVIP contrast estimate and change in behavioral performance from baseline to post-treatment within the fatigued cohort for (A)** Left Parietal and **(B)** Right Parietal regions of interest.

Within the fatigued sample, the change in RVIP task-related activity from baseline to post-treatment was pursued in order to assess the relationship with changes in behavioral performance. Positive correlations were observed between the change in RVIP task performance from baseline to post-treatment, as indexed by total number of correct target detections, and change in RVIP contrast estimate from baseline to post-treatment in the Left Parietal (*r*_s_ = 0.681, *p* = 0.015), Right Parietal (*r*_s_ = 0.628, *p* = 0.029) and SMA (*r*_s_ = 0.491, *p* = 0.105) regions of interest. Increased functional brain activity during RVIP task completion at follow-up was found to be associated with more improved performance amongst fatigued participants. Scatterplots of the association between change in functional activity and behavioral performance are shown in **Figure 2** for the two parietal regions of interest, in which significant (*p* < 0.05) correlations were observed.

### SSVEP

#### A-X CPT: Baseline SSVEP Task Effects

**FIGURE 3:**
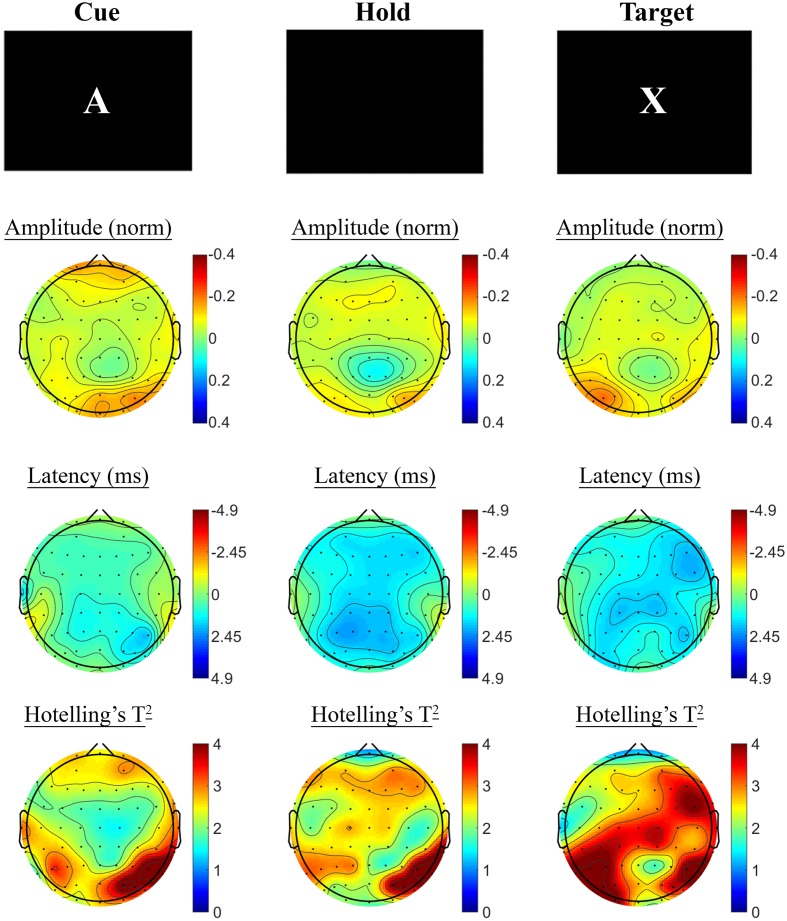
**A-X CPT SSVEP amplitude and phase differences, contrasted with the control task, at the baseline assessment across the sample.** Columns show topographic maps of SSVEP amplitude (top) and phase (as latency, middle), with Hotelling’s T^2^ corresponding to the contrast of active and control task variants for Cue **(Left)**, Hold **(Middle)** and Target **(Right)** task segments.

SSVEP amplitude and phase differences between active and control task variants across the sample at baseline are shown in **Figure 3**. Consistent with previous research, reduced amplitude was observed across the three periods of the task, in prefrontal and posterior sites during the Cue period, continuing across fronto-central and posterior sites during Hold and Target components of the task. SSVEP phase demonstrated modest frontal advance during the Cue period, whilst a right posterior phase lag during this period extended more broadly across Hold and Target periods. The prefrontal phase advance previously reported in combination with amplitude reduction during Hold and Target task components (cf. Silberstein et al., 1998; Silberstein et al., 2000; White et al., 2016) was less apparent in the current sample.

#### A-X CPT: Treatment Group SSVEP Analysis

**FIGURE 4:**
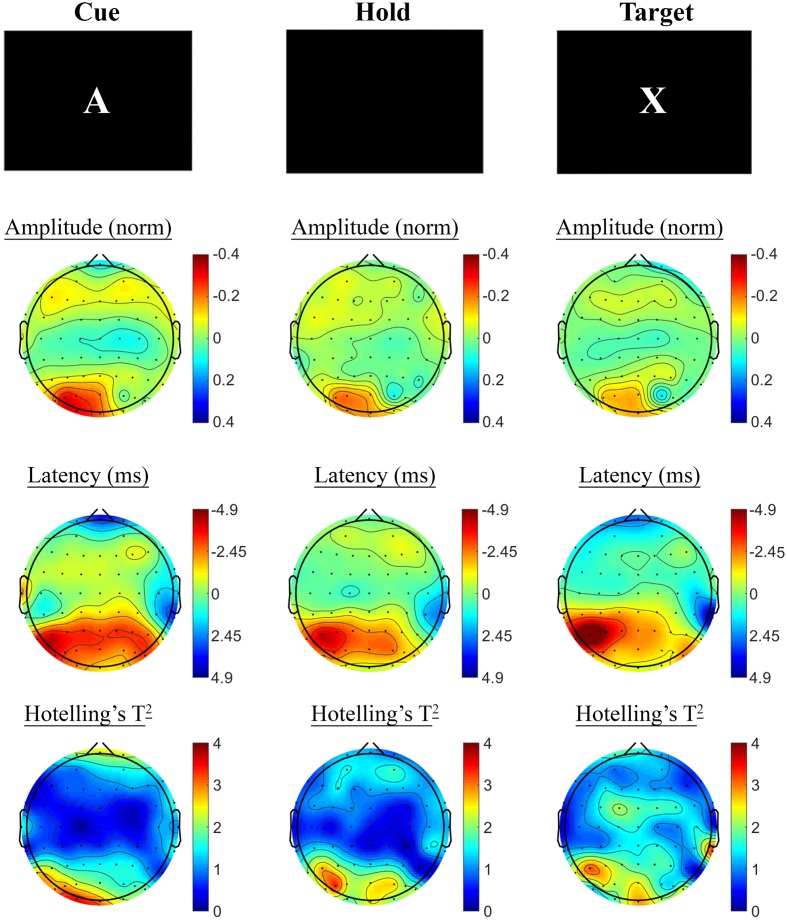
**A-X CPT SSVEP amplitude and phase changes from baseline to post-treatment in the placebo group.** Columns show topographic maps of SSVEP amplitude (top) and phase (as latency, middle), with Hotelling’s T^2^ corresponding to the contrast of active and control task variants for Cue **(Left)**, Hold **(Middle)**, and Target **(Right)** task segments.

**FIGURE 5:**
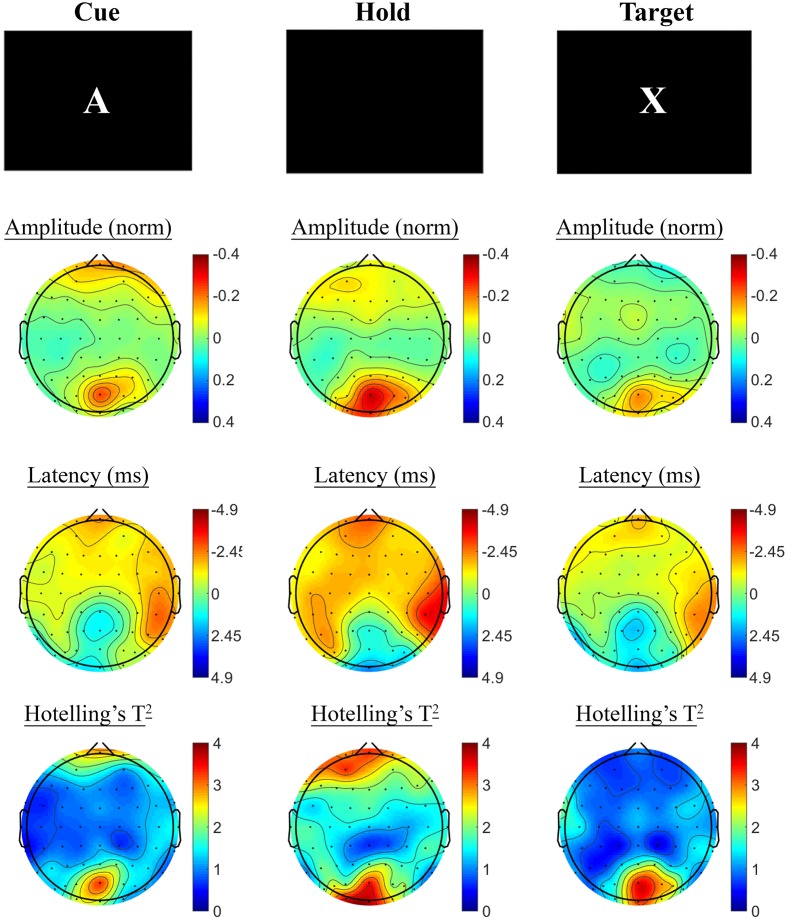
**A-X CPT SSVEP amplitude and phase changes from baseline to post-treatment in the active MVM treatment group.** Columns show topographic maps of SSVEP amplitude (top) and phase (as latency, middle), with Hotelling’s T^2^ corresponding to the contrast of active and control task variants for Cue **(Left)**, Hold **(Middle)** and Target **(Right)** task segments.

Changes in SSVEP response from baseline to post-treatment were explored for placebo and active MVM treatment groups separately. For the placebo treatment group, there were no significant differences in SSVEP response to the A-X CPT between baseline and post-treatment visits across the three task components (shown in **Figure 4**). Trends (*p* < 0.05, not reaching threshold) were observed for a small number of parieto-occipital electrodes where amplitude reduction and phase advance across the three task components were observed at follow-up assessment. The active MVM treatment group showed significant amplitude reduction post-treatment in a single occipital site during the Hold period (shown in **Figure 5**). In addition, a cluster of frontal electrodes showed a trend (*p* < 0.05) toward decreased amplitude and phase advance. While this pattern of frontal phase advance and amplitude reduction did not reach significance when adjusting for multiple comparisons, this effect is consistent, both in terms of task component and electrode locations, to previously reported changes in SSVEP response during completion of the A-X CPT after an acute dose of the same MVM intervention (White et al., 2016). Patterns of change and statistical significance were unchanged when sensitivity analyses were pursued, excluding behavioral outliers. There were no significant correlations between the change in behavioral performance from baseline to post-treatment and these changes in SSVEP response during A-X CPT.

#### Spatial Working Memory: Baseline SSVEP Task Effects

**FIGURE 6:**
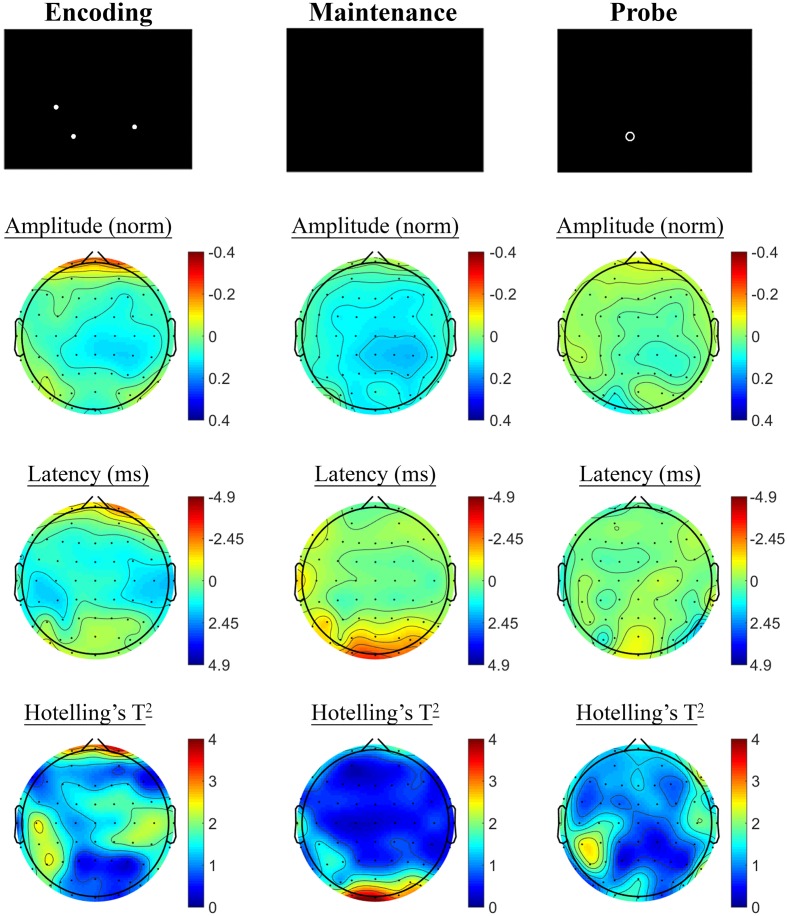
**Spatial Working Memory SSVEP amplitude and phase differences, contrasted with the control task, at the baseline assessment across the sample.** Columns show topographic maps of SSVEP amplitude (top) and phase (as latency, middle), with Hotelling’s T^2^ corresponding to the contrast of active and control task variants for Encoding **(Left)**, Maintenance **(Middle)**, and Retrieval **(Right)** task segments.

The SSVEP response to the active task variant contrasted with the control variant showed a pattern of reduced SSVEP amplitude and phase advance in prefrontal electrodes during encoding, followed by posterior phase advance during the hold period, as shown in **Figure 6**. No significant differences were observed in the retrieval segment, in the first 1000 ms following presentation of the probe, between the control and active task variants.

#### Spatial Working Memory: Treatment Group SSVEP Analysis

**FIGURE 7:**
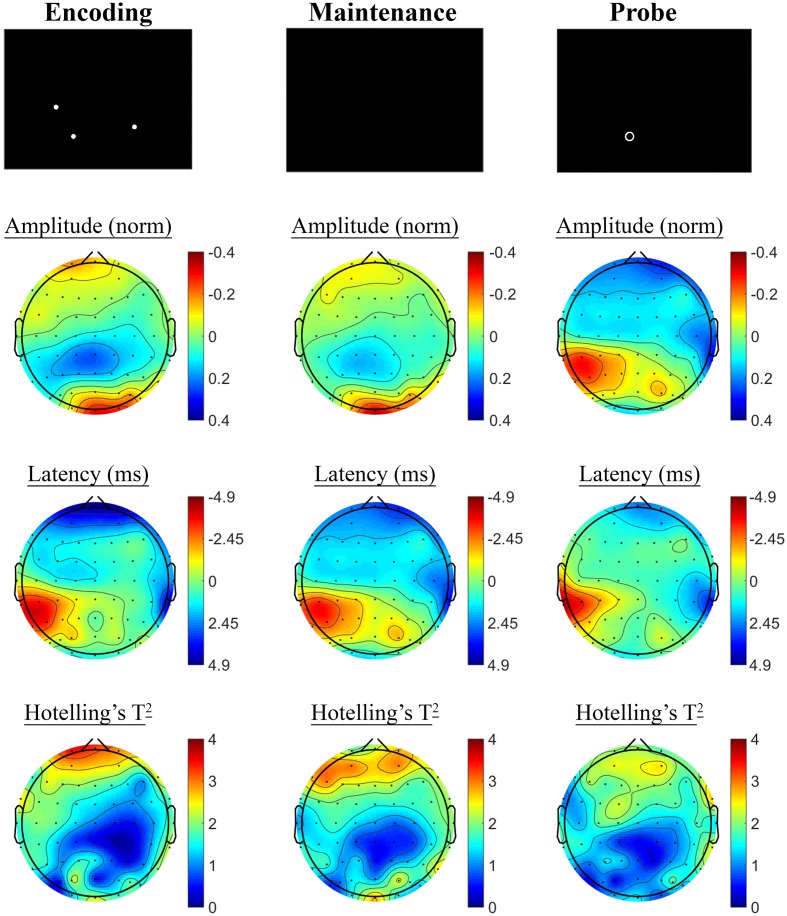
**Spatial Working Memory SSVEP amplitude and phase changes from baseline to post-treatment in the placebo treatment group.** Columns show topographic maps of SSVEP amplitude (top) and phase (as latency, middle), with Hotelling’s T^2^ corresponding to the contrast of active and control task variants for Encoding **(Left)**, Maintenance **(Middle)** and Retrieval **(Right)** task segments.

**FIGURE 8:**
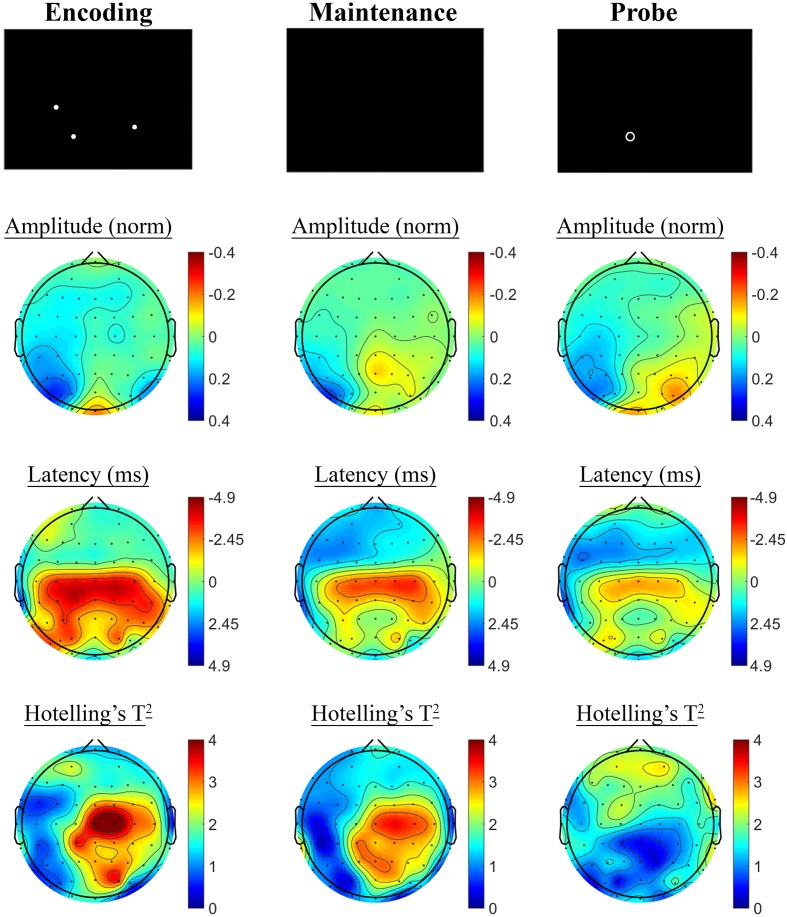
**Spatial Working Memory SSVEP amplitude and phase changes from baseline to post-treatment in the active MVM treatment group.** Columns show topographic maps of SSVEP amplitude (top) and phase (as latency, middle), with Hotelling’s T2 corresponding to the contrast of active and control task variants for Encoding **(Left)**, Maintenance **(Middle)** and Retrieval **(Right)** task segments.

Contrasting SSVEP responses post-treatment with baseline activity during the Spatial Working Memory task showed no significant changes in the placebo treatment group, but evidence of significant changes post-treatment in the active MVM treatment group. In the placebo group, shown in **Figure 7**, trends toward greater phase lag (increased latency) were observed in the three pre-frontal electrodes during the encoding period, in addition to a left frontal pattern of phase lag and amplitude reduction in the maintenance period, though neither reached significance when adjusting for multiple comparisons. The active MVM treatment group showed centro-parietal SSVEP phase advance (reduced latency) during the encoding segment of the Spatial Working Memory task (shown in **Figure 8**), reaching criteria for significance in two central electrodes when adjusting for multiple comparisons. This pattern of SSVEP phase advance in centro-parietal regions persisted into the maintenance period, though it did not reach criteria for significance in this time window. This change in SSVEP phase response in central electrodes during encoding was not correlated with the change in behavioral performance from baseline to post-treatment within the active MVM treatment group.

## Discussion

This study investigated the effects of 4 weeks daily treatment with a MVM preparation on functional brain activity, as assessed by complementary hemodynamic and electrophysiological methods. Both fMRI and SSVEP modalities revealed changes in functional brain activity following treatment with the active MVM treatment, with converging patterns of activity consistent with greater activity in overlapping centro-parietal regions during working memory task performance. Within the fMRI data, this increase in task-related BOLD response was correlated with improved behavioral performance, however, these fMRI findings were only apparent amongst the fatigued cohort of participants who had first undergone SSVEP assessment. The active MVM treatment was also associated with greater posterior SSVEP amplitude reductions post-treatment during the period between the cue and target stimuli of the A-X CPT, as well as a trend toward SSVEP amplitude reductions and phase advance across frontal regions. MVM treatment was not associated with significant changes in behavioral performance on the cognitive tasks completed during monitoring of functional brain activity. Thus, outcomes of this trial point to changes in functional brain activity associated with MVM treatment over 4 weeks. These findings, however, must be considered in light of the limited evidence of behavioral change in cognitive task performance (while acknowledging that the cognitive evaluation were primarily designed as activation tasks rather than to capture any treatment-related effects).

### Changes in Functional Brain Activity during Working Memory

Changes in functional brain activity reflecting greater activity in centro-parietal regions in the MVM arm were observed during working memory tasks using both imaging methodologies. As part of the SSVEP changes, the active MVM treatment group showed SSVEP phase advance (reduced latency) across centro-parietal regions. The temporal resolution of the SSVEP method allows isolation of sub-components of the working memory task, which indicated this increase was maximal during the encoding period of the task. This pattern was broadly consistent with the effects observed in the fatigued cohort during the RVIP task using fMRI, where bilateral parietal and supplementary motor area regions of interest showed greater task-related BOLD in the MVM group. While these two tasks represent distinct working memory paradigms (delayed matching-to-sample versus continuous performance), the probing of treatment effects within the fMRI data used contrast images derived from the active task minus the control task variant. This allows relatively complete isolation of working memory demands during the active task. Thus these two patterns of change provide converging evidence of increased functional brain activity associated with MVM supplementation during actively engaged working memory processes.

Given the preliminary nature of this investigation, the source of disparate findings relating to potential treatment-related changes in task-related BOLD response between the fatigued and non-fatigued fMRI cohorts remains unclear. One possible explanation is that, amongst healthy adults, the neurocognitive benefits of this supplementation are more apparent under conditions of greater cognitive demand. Supporting such a possibility, previous work exploring cognitive changes following 4 weeks of MVM supplementation in healthy adult males demonstrated benefits, both in terms of cognitive performance and subjective ratings of fatigue, during completion of a 1 h cognitive demand battery (Kennedy et al., 2010). Given the relatively insensitive approach to detecting BOLD changes adopted in the current investigation (extracting mean task-related estimates of activity from broad regions of interest), it may be that robust differences are only apparent when placing the individual under greater demand. Further replication and detailed investigation of this effect is required to better understand these effects.

The observed changes in SSVEP response during completion of the spatial working memory task associated with MVM supplementation are in contrast with those of Macpherson et al. (2012), where latency increases (phase lag) were observed in elderly women after MVM supplementation during the retrieval period of the same task used herein. However, the previous trial by Macpherson et al. (2012) studied an intervention which contained 19 plant extracts and three probiotics in addition to micronutrients. For example, the intervention described by Macpherson et al. (2012) included a relatively large dose of *Ginkgo Biloba* (1000 mg), an extract which has been shown to exert a range of complex actions *in vitro* as a GABA, glycine and 5HT receptor antagonist (Huang et al., 2003; Hawthorne et al., 2006; Thompson et al., 2011). As such, the disparate findings of Macpherson et al. (2012) and those of the present report, in which treatment involved only B vitamins, vitamin C, and three minerals, are likely driven by the variability in the treatment constituents.

### Changes in Functional Brain Activity during Cued Attention

The active MVM treatment group also showed greater posterior SSVEP amplitude reductions post-treatment during the period between the cue and target stimuli of the A-X task, as well as a trend of reduced amplitude and phase advance across frontal regions. Previous studies to explore SSVEP correlates of attention showed decreased amplitude in posterior electrodes where task demands were increased during visual vigilance tasks (Silberstein et al., 1990), which are consistent with the pattern of results observed in the present study across all participants at baseline. This reduced SSVEP amplitude during attention tasks which require ongoing processing of visual stimuli is thought to reflect an inhibition of the re-entrant cortico-cortical loop transmission efficiency through cortical layer I, as the sensory inputs to layer IV are enhanced with visual processing of the task demands (Silberstein et al., 2001). At this stage, it is unclear whether the reduced posterior SSVEP amplitude in this window following the cue stimuli reflects this visual processing, or a greater top-down biasing of attention to the visual stimuli following the cue in a manner paralleling the proposed role for alpha oscillations in top-down control of attention (for a review, see Foxe and Snyder, 2011). Similarly, the potential mechanism by which this amplitude was further reduced post-MVM treatment is not fully elucidated.

We have previously reported a single dose of MVM resulted in frontal SSVEP amplitude reductions and phase advance, in this period between cue and target stimuli during the A-X CPT (White et al., 2016). Whilst not reaching criteria for significance when controlling for multiple comparisons, the frontal SSVEP amplitude reduction and phase advance observed in the current trial was consistent with this previous acute investigation off MVM treatment, in terms of the precise task window, the direction of change in both SSVEP amplitude and phase, and the spatial location of the effect. The previous study, however, observed that this pattern of change was correlated with the change in task performance, this was not the case in the current study.

### Behavioral Measures of Cognitive Task Performance

Despite evidence for changes in functional brain activity, behavioral performance on the cognitive tasks completed during monitoring of functional brain activity did not significantly differ between treatment groups in parallel with these neurophysiological changes. This trial was designed as an exploratory investigation with functional brain activity (measured with SSVEP and fMRI) as the primary outcomes. As such, *a priori* sample size determination did not power the study to detect behavioral performance changes, nor were the task paradigms themselves optimized for capturing behavioral differences (rather they were operationalized primarily as activation tasks). Studies to test cognitive benefits of MVM supplementation in healthy adults have involved considerably larger samples in detecting relatively subtle cognitive effects (Haskell et al., 2010; Kennedy et al., 2010), indeed, meta-analysis suggests a standardized mean difference for immediate recall in the small to medium range (Grima et al., 2012), further emphasizing the need for considerably larger sample sizes where assessing cognitive performance changes as the primary outcome.

An alternative strategy in assessing the functional relevance of observed neurophysiological changes associated with MVM treatment has been to explore the association between the change in functional brain activity and the change in behavioral measures of cognitive task performance. In the present study, the greater RVIP task-related BOLD response observed after active MVM treatment within the fatigued cohort was correlated with performance changes. This indicates that the greater the increase in functional brain activity post-treatment, the more improved behavioral performance on the RVIP task. However, while we have previously reported associations between patterns of SSVEP response change and behavioral performance (White et al., 2016), this relationship was not observed in the current sample, despite trends toward faster response times on the A-X CPT in the MVM treatment group compared to placebo. It remains to be fully elucidated by larger scale research whether the observed neurophysiological effects of MVM administration represent changes of relevance to neurocognitive function which were subthreshold given the limitations in sample size and insensitivity of the behavioral cognitive outcomes in the present study.

### Mechanisms of MVM Supplementation in Neurocognitive Function

The very nature of MVM supplementation complicates the isolation of specific mechanisms by which these interventions exert observed effects, given both the multitude of constituents and the multitude of cellular processes in which they serve as critical cofactors (Kennedy, 2016). Indeed, the complementary and interactive roles these micronutrients play in such critical cellular pathways suggest that a reductionist search to isolate a single candidate active constituent and pathway may be inappropriate (Kennedy (2016) for a thorough account of the potential advantages of multiple micronutrient administration over single nutrient preparations). Instead a more integrated view of the multiple mechanisms impacted by MVM treatment may be relevant. For example, the role of these micronutrients in energy metabolism and neurotransmitter synthesis appear as two prominent, not mutually exclusive, pathways through which MVM supplementation may exert neurocognitive effects such as the improved mood (White et al., 2015) and potential changes to functional brain activity during cognitive task engagement observed as part of the current trial.

Nonetheless, MVM formulations are not standardized in the marketplace and greater provision of one nutrient relative to another may contribute to the benefits obtained from supplementation. In the current study, the levels of B vitamins are at or above the recommended daily intakes (**Table 1**), suggesting that such treatment should redress any insufficiencies in these micronutrients (White et al., 2015). Blood markers showed that levels of key vitamins did change in this cohort (White et al., 2015), although the study was not powered to relate individual differences in nutrient status to neurocognitive or behavioral responses. Future studies may also consider the influence of pre-existing health and nutrient status of the individuals receiving the supplement. This is particularly important given evidence of inadequate intakes of a range of micronutrients associated with a Western style diet (Troesch et al., 2012).

Beyond the theoretical biochemical pathways which link micronutrient treatment with these mechanisms, experimental data also supports the possibility for manipulating these processes through micronutrient administration. Daily MVM supplementation amongst obese adult females over 26 weeks has been shown to result in increased resting energy expenditure, along with reductions in fat mass, body weight and cholesterol (Li et al., 2010). Furthermore, Kennedy et al. (2016) reported increased energy expenditure following both acute and chronic MVM administration in healthy younger adult females during completion of a cognitive battery. In support of the notion that administration of even a single B vitamin may influence neurotransmitter synthesis, there exists research in lower mammals demonstrating that administration of vitamin B6 leads to an increase in central serotonin levels (Hartvig et al., 1995; Calderon-Guzman et al., 2004).

Whilst the present work has focused on enhancing function in healthy adults, a further pathway through which a more targeted B vitamin supplementation scheme may exert neurocognitive changes in at least one at-risk population warrants discussion. Smith et al. (2010) have described a very clear candidate mechanism by which B vitamin supplementation may exert neurocognitive changes, reducing homocysteine as part of the methionine cycle, in turn minimizing gray matter atrophy and slowing cognitive decline in older adults diagnosed with Mild Cognitive Impairment presenting with elevated homocysteine (de Jager et al., 2012; Douaud et al., 2013). Thus, while further research is required to fully characterize the physiological changes associated with MVM supplementation in both health and disease, there is evidence to suggest energy metabolism, neurotransmitter synthesis, as well as direct outcomes of one carbon transfer cycles such as reduced homocysteine, are mechanisms modifiable by micronutrient administration.

## Conclusion

An emerging focus on optimal micronutrient intake, beyond that required to avoid clinical deficiency, questions whether otherwise healthy individuals may gain benefit from MVM supplementation. Whilst some research supports benefits to mood and possibly cognitive function, research exploring parallel physiological changes is also underway. Outcomes of the present trial provide evidence to suggest potential changes in functional brain activity following 4 weeks MVM supplementation using both hemodynamic and electrophysiological measures. In particular, converging evidence from fMRI and SSVEP recordings showed patterns of brain activity consistent with greater activity in overlapping centro-parietal regions following MVM administration during working memory. The fMRI findings were, however, only observed amongst the subset of participants undergoing this assessment under fatigue. Evidence of SSVEP changes during a cued attention task were also observed, partially consistent with a previous report after a single dose (White et al., 2016), however, these changes were not associated with behavioral performance on the task. All of the observed changes were consistent with recruitment of additional neural resources or greater excitatory processes during task engagement, that is, greater task-related BOLD response and SSVEP amplitude reductions and phase advance. Thus, the present study provides preliminary evidence to suggest daily ingestion of a high dose B vitamin micronutrient supplement over 4 weeks may increase task-related functional brain activity in healthy adults. These findings are of further relevance given that, while a healthy diet may be sufficient to maintain adequate supplies of these essential micronutrients, dietary surveys of countries which follow a modern Western dietary pattern indicate a significant proportion of the population may not meet recommendations for a range of micronutrients, including B vitamins (Troesch et al., 2012).

## Author Contributions

AS and DW conceived the study and had significant input into design and interpretation (aided by AP). KC and RP were largely responsible for data collection and interpretation. DW, MH and RP were responsible for data processing. All authors had input into the final manuscript.

## Conflict of Interest Statement

DW, AP, and AS have received research funding, consultancy and speaker fees from the food and nutrition industry, including Bayer, who funded the study. Bayer AG had no role in the design of the study; in the collection, analysis or interpretation of data; in the writing of the manuscript; nor in the decision to publish the results. The reviewer NAK and handling Editor declared their shared affiliation, and the handling Editor states that the process nevertheless met the standards of a fair and objective review.

## Footnotes

1 www.neurobs.com

2 http://marsbar.sourceforge.net
