# Supplementary material for: Functional Brain Activity Changes after 4 Weeks Supplementation with a Multi-Vitamin/Mineral Combination: A Randomized, Double-Blind, Placebo-Controlled Trial Exploring Functional Magnetic Resonance Imaging and Steady-State Visual Evoked Potentials during Working Memory
Source: Front Aging Neurosci. 2016 Dec 2;8:288. doi: 10.3389/fnagi.2016.00288 (PMC5133263; doi:10.3389/fnagi.2016.00288)
Supplement: Supplementary file 1 [file Data_Sheet_1.DOCX]

Supplementary Material

Functional brain activity changes after four weeks supplementation with a multi-vitamin/mineral combination: A randomized, double-blind, placebo-controlled trial exploring functional Magnetic Resonance Imaging and Steady-State Visual Evoked Potentials during working memory.

David J. White, Katherine H.M. Cox, Matthew E. Hughes, Andrew Pipingas, Riccarda Peters, and Andrew B. Scholey*

*** Correspondence:** Centre for Human Psychopharmacology, School of Health Sciences, Swinburne University, Mail H24 PO Box 218, Hawthorn, VIC, 3122, Australia.

andrew@scholeylab.com

# Defining Regions of Interest

An independent sample of healthy young adults completing the identical version of the RVIP task implemented as part of the intervention study were used to define the ROIs in which potential neurocognitive effects of the intervention were explored. The sample consisted of 13 healthy young adults (8 Females, Mean age = 22.5 years) matching eligibility criteria for the intervention trial (right handed, non-smokers, no history of psychiatric or neurological diagnosis). All data acquisition, pre-processing, and task modelling was identical to that described in the Methods section. Contrast images obtained from subtracting the Active task blocks from the Control blocks were entered into a second-level model, with a random effects one-sample t-test used to identify regions significantly greater during the Active blocks compared to Control blocks using SPM8 software (Wellcome Trust Centre for Neuroimaging, London, UK). Statistic parametric maps were thresholded using a FWE-corrected *p*<.05 at the cluster level, using a cluster defining threshold of *p*<.001 (T>3.93). Details of the five significant clusters are provided in Supplementary Table 1 below, and in Supplementary Figure 1. These five clusters subsequently formed the regions of interest used to study the impact of supplementation.

**Supplementary Table 1.** Details of the five significant clusters contrasting Active > Control blocks during completion of the RVIP task. These clusters comprised the ROIs for exploring intervention-related changes in functional activity.

| Cluster *p* | Cluster size | Peak | MNI co-ord  (centre of mass) | | | Anatomical Region |
| --- | --- | --- | --- | --- | --- | --- |
| (FWE-corr) | *k* voxels | *t* | x | y | z |  |
| <.001 | 1884 | 12.18 | -27 | -64 | 44 | L Inf/Ang/Sup Parietal Gyri |
| <.001 | 1871 | 10.03 | 31 | -60 | 46 | R Inf/Supramarg/Sup Parietal Gyri |
| <.001 | 716 | 6.63 | -48 | 5 | 31 | L Middle Frontal Gyrus |
| <.001 | 579 | 6.83 | 0 | 8 | 58 | B Supplementary Motor Area |
| 0.003 | 441 | 10.26 | -35 | 20 | 1 | L Insula / Inf Orbito-Frontal |


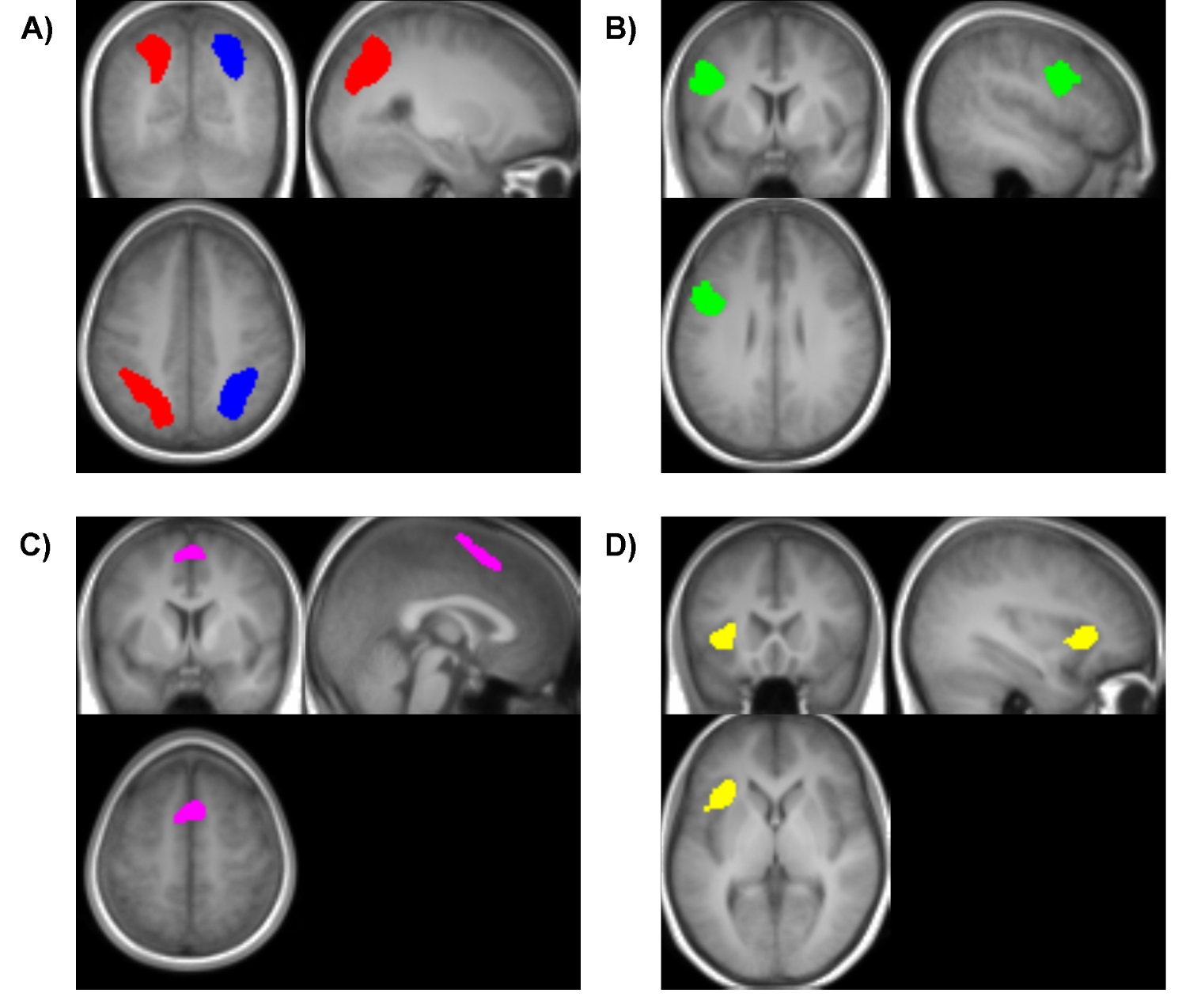


**Supplementary Figure 1.** The five regions of interest utilized for assessing intervention effects during RVIP task completion, defined using an independent sample of healthy adults, showing (A) left (red) and right (blue) parietal clusters, (B) left middle frontal, (C) supplementary motor area, and (D) left insula, plotted onto mean normalized T1 image from study sample.

# fMRI task effects at baseline

Baseline visit patterns of task-related activity were studied in order to confirm that performance of the RVIP and IT tasks elicited patterns of brain activity consistent with previous research, also justifying the regions of interest identified from the independent sample for use in exploring treatment-related effects during RVIP task performance. Collapsing across treatment groups, whole-brain random effects analyses were conducted, using a FWE-corrected p<.05 at the cluster level, with a cluster defining threshold of p<.001. For the RVIP task, the active task > control task contrast image formed the input for this analysis, whilst the statistical parametric map resulting from the (linear) parametric modulation of BOLD response by stimulus presentation length was used to explore task effects for the IT task. The anatomical labels for significant clusters was determined using the Automated Anatomical Labelling atlas [1], within the WFU Pickatlas toolbox [2].

## Inspection Time task

Stimulus presentation length formed the input as a parametric modulator in the first level modelling of IT task effects. This regressor, highest for the longest presentation length, is orthogonalised with respect to the trial onset regressor, for correct trials only, as such the parameter estimate for this parametric modulator expresses how well BOLD fluctuations not explained by the average response to all stimulus onsets covaries with stimulus presentation length.

A model was run for fatigued participants and non-fatigued participants separately. Two participants did not have behavioral results due to a technical fault, and as such were excluded from this analysis as trials with incorrect responses are typically excluded from analysis of functional imaging correlates of inspection time [3, 4], and a further four participants were excluded as performance across all stimulus durations was around chance levels. As a result, 13 participants were included in each fatigued and non-fatigued groups.

No significant positive or negative parametric modulation of BOLD signal were observed in either fatigued or non-fatigued groups (FWE-corrected p<.05 at the cluster level). In the absence of reliable task effects at baseline, this task was not pursued further for the impact of treatment.

## Rapid Visual Information Processing task

Consistent with previous fMRI investigations of the RVIP task, baseline data showed significantly greater BOLD response during the active task variant across bilateral parietal, occipital, cerebellar and frontal regions. One participant in the fatigued group was excluded from analysis, due to poor behavioral performance.

Two large clusters, one anterior and one posterior, showed significantly greater BOLD response to the active RVIP task variant compared to the control variant within both fatigued and non-fatigued samples. These clusters are summarized in Supplementary Table 2 and shown in Supplementary Figure 2 below. The posterior cluster included bilateral inferior and superior parietal gyri, in addition to inferior temporal, occipital and cerebellar regions. The anterior cluster comprised large sections of bilateral frontal cortex, including precentral, inferior, middle and superior frontal gyri, supplementary motor area, in addition to thalamus, insula and basal ganglia.

a)**
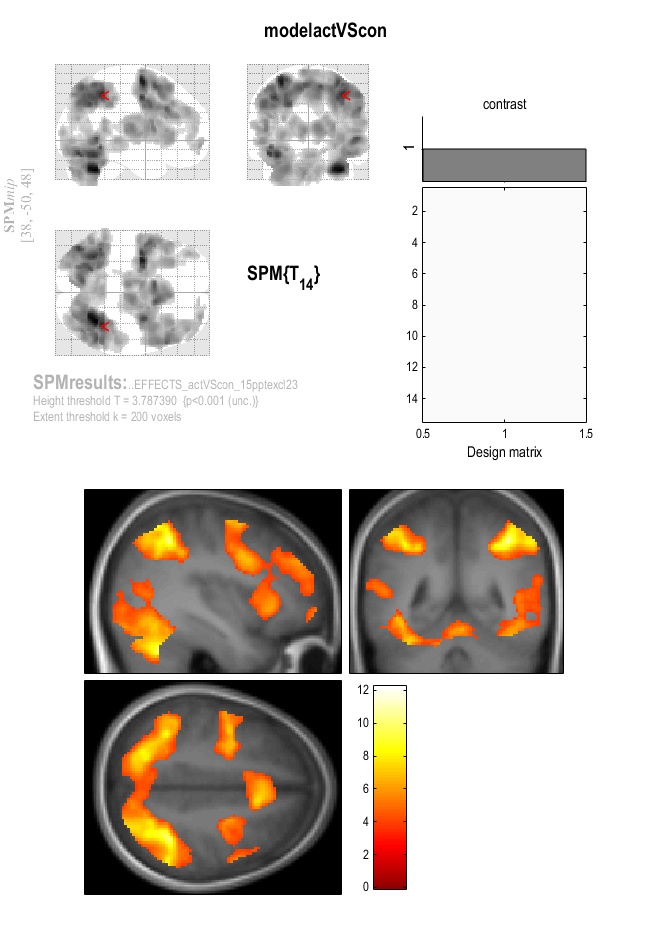
**b)**
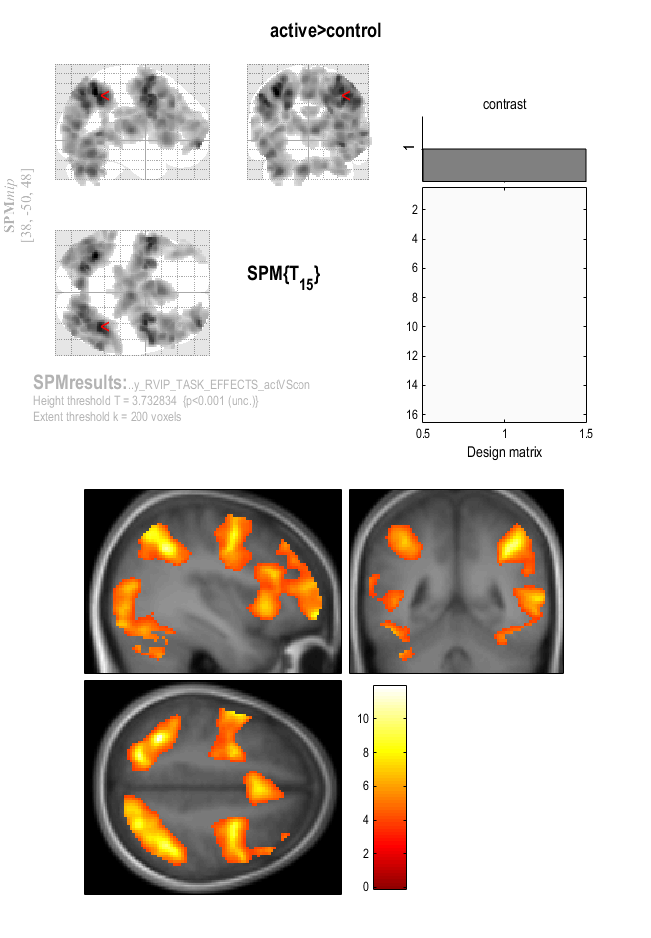
**

**Supplementary Figure 2.** Statistic parametric map showing result of contrasting active task versus control for (a) the fatigued sample and (b) the non-fatigued sample at baseline, mapped onto mean normalized T1-weighted image from the 32 study participants. Section through right parietal peak (MNI: 38, -50, 48), T-statistics thresholded at FWE p<.05 at the cluster level (cluster defining threshold T>3.79).

**Supplementary Table 2.** Anatomical regions comprising the two large clusters showing significant BOLD response during the active RVIP task amongst fatigued and non-fatigued samples.

|  |  | Fatigued sample  Posterior k = 19431  Anterior k = 16947 | | Non-fatigued sample  Posterior k = 17252  Anterior k = 20011 | |  |
| --- | --- | --- | --- | --- | --- | --- |
| Cluster | AAL region | Left hemisphere (voxels) | Right hemisphere (voxels) | Left hemisphere (voxels) | Right hemisphere (voxels) | |
| Posterior | Inferior Parietal | 1052 | 872 | 1125 | 702 | |
|  | Superior Parietal | 952 | 784 | 835 | 699 | |
|  | Cerebellum (combined) | 2989 | 1401 | 2014 | 1051 | |
|  | Inf/Mid Temporal + Fusiform | 1529 | 2369 | 1653 | 1871 | |
|  | Occipital (combined) | 1850 | 1732 | 2141 | 2422 | |
|  |  |  |  |  |  | |
| Anterior | Precentral Frontal | 1663 | 897 | 1924 | 1259 | |
|  | Middle Frontal | 700 | 1429 | 852 | 2339 | |
|  | Inferior Frontal | 1064 | 2027 | 783 | 2049 | |
|  | SMA | 881 | 889 | 840 | 702 | |
|  | Thalamus | 589 | 681 | 601 | 676 | |
|  | Basal Ganglia | 496 | 624 | 206 | 498 | |
|  | Insula | 428 | 434 | - | 475 | |
|  |  |  |  |  |  | |

Notes: SMA=Supplementary Motor Area

# Participant Flow Diagram

Supplementary Figure 3 below shows a CONSORT style Flow Diagram detailing progression of participants through the trial.


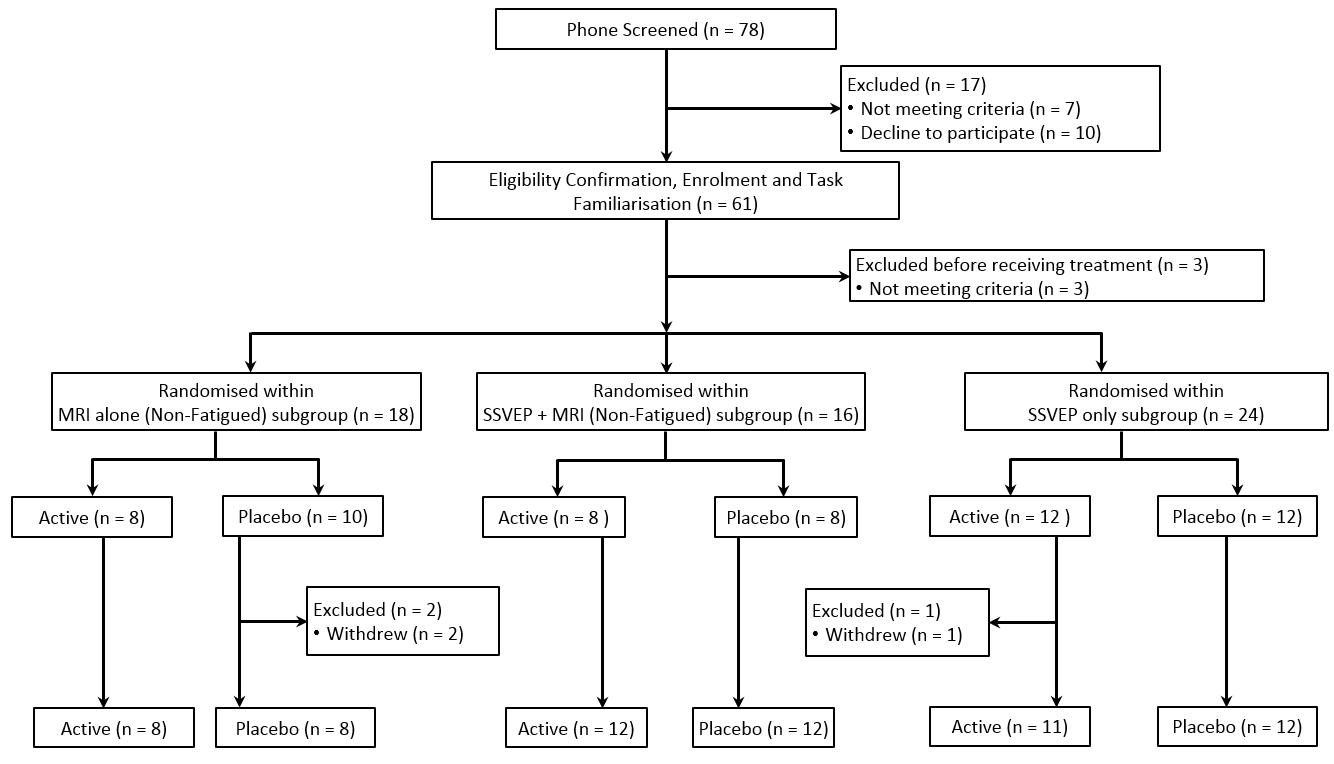


Supplementary Figure 3. Flow of participants through the trial, showing the three testing streams. Figure adapted from White et al. (2015).

Supplementary References

1. Tzourio-Mazoyer, N., et al., *Automated anatomical labeling of activations in SPM using a macroscopic anatomical parcellation of the MNI MRI single-subject brain.* Neuroimage, 2002. **15**(1): p. 273-289.

2. Maldjian, J.A., et al., *An automated method for neuroanatomic and cytoarchitectonic atlas-based interrogation of fMRI data sets.* Neuroimage, 2003. **19**(3): p. 1233-9.

3. Deary, I.J., et al., *The functional anatomy of inspection time: an event-related fMRI study.* Neuroimage, 2004. **22**(4): p. 1466-79.

4. Waiter, G.D., et al., *Is retaining the youthful functional anatomy underlying speed of information processing a signature of successful cognitive ageing? An event-related fMRI study of inspection time performance.* Neuroimage, 2008. **41**(2): p. 581-95.
